# Limited host availability disrupts the genetic correlation between virulence and transmission

**DOI:** 10.1093/evlett/qrac008

**Published:** 2023-01-31

**Authors:** Diogo P Godinho, Leonor R Rodrigues, Sophie Lefèvre, Laurane Delteil, André F Mira, Inês R Fragata, Sara Magalhães, Alison B Duncan

**Affiliations:** cE3c: Centre for Ecology, Evolution, and Environmental Changes, Faculty of Sciences, University of Lisbon, Lisboa, Portugal; cE3c: Centre for Ecology, Evolution, and Environmental Changes, Faculty of Sciences, University of Lisbon, Lisboa, Portugal; Institut des Sciences de l’Évolution, Université de Montpellier, CNRS, IRD, EPHE, Montpellier, France; Institut des Sciences de l’Évolution, Université de Montpellier, CNRS, IRD, EPHE, Montpellier, France; cE3c: Centre for Ecology, Evolution, and Environmental Changes, Faculty of Sciences, University of Lisbon, Lisboa, Portugal; cE3c: Centre for Ecology, Evolution, and Environmental Changes, Faculty of Sciences, University of Lisbon, Lisboa, Portugal; Departamento de Biologia Animal, Faculdade de Ciências da Universidade de Lisboa, Lisboa, Portugal; cE3c: Centre for Ecology, Evolution, and Environmental Changes, Faculty of Sciences, University of Lisbon, Lisboa, Portugal; Departamento de Biologia Animal, Faculdade de Ciências da Universidade de Lisboa, Lisboa, Portugal; Institut des Sciences de l’Évolution, Université de Montpellier, CNRS, IRD, EPHE, Montpellier, France

**Keywords:** host-parasite interactions, spider, mites, inbred lines, trade, off

## Abstract

Virulence is expected to be linked to parasite fitness via transmission. However, it is not clear whether this relationship is genetically determined, nor if it differs when transmission occurs continuously during, or only at the end of, the infection period. Here, we used inbred lines of the macroparasitic spider mite *Tetranychus urticae* to disentangle genetic vs. nongenetic correlations among traits, while varying parasite density and opportunities for transmission. A positive genetic correlation between virulence and the number of transmitting stages produced was found under continuous transmission. However, if transmission occurred only at the end of the infection period, this genetic correlation disappeared. Instead, we observed a negative relationship between virulence and the number of transmitting stages, driven by density dependence. Thus, within-host density dependence caused by reduced opportunities for transmission may hamper selection for higher virulence, providing a novel explanation as to why limited host availability leads to lower virulence.

## Introduction

Virulence, the harm inflicted by parasites on their hosts, is a trait with high relevance for human, animal, plant, and ecosystem health. Parasites replicate within their host, exploiting their resources and damaging tissues, thus virulence is often positively correlated with replication rate (reviewed in Acevedo et al., 2019). Thus, it is expected that higher replication rates lead to more parasites emerging to infect novel hosts, with a positive impact on parasite fitness (i.e., the reproductive growth rate, *R*_0_; Anderson & May, 1982). Therefore, selection is expected to also favor higher virulence. This holds as long as virulence is not so high as to induce host mortality, in which case higher virulence shortens the infection period, reducing the chances for transmission, or to cause host morbidity, decreasing the encounter rate with uninfected hosts, which will also hamper transmission. This reduction in transmission opportunities decreases parasite fitness, leading to the so-called virulence-transmission trade-off (Anderson & May, 1982).

This relationship between virulence and parasite fitness is an assumption of most theoretical models of virulence evolution (Anderson & May, 1982; Frank, 1996; Wild et al., 2009). Because evolution rests on the existence of genetic variation, it is implicit that the underlying correlation between virulence and transmission is genetic. However, although such correlations have been found in several systems (reviewed in Acevedo et al., 2019), most studies used parasite isolates with different geographic origins and thus that differ not only genetically but also in their recent ecological and evolutionary history (de Roode et al., 2008; Doumayrou et al., 2013; Ebert, 1994; Mackinnon & Read, 1999). Hence, values for virulence and transmission may hinge upon different environments selecting for particular combinations of traits, and not on hardwired genetic correlations among traits (Hochberg, 1998). Disentangling between the environmental and genetic factors affecting the relationship between virulence and transmission is possible by using inbred lines derived from the same genetically diverse parasite population (Godinho et al., 2020). Using this approach is key to identify the conditions under which these parasite traits can evolve independently.

The evolution of virulence is also expected to be contingent upon other factors such as host population structure (Thrall & Burdon, 2003) and the timing of transmission (Day, 2003). Theory predicts that virulence should be reduced in viscous populations (in which individuals tend to remain in the patch where they were born) because in these populations relatedness among parasites increases and the benefits of producing dispersers are reduced (Boots & Sasaki, 1999; Lion & Boots, 2010; Wild et al., 2009). Experimental evidence for this prediction has been shown in phages (Kerr et al., 2006) and viruses (Boots & Mealor, 2007). Selection on virulence is also predicted to be contingent upon the timing of transmission, with early transmission selecting for higher virulence (Day, 2003), which has been shown empirically in microcosm populations (Berngruber et al., 2013). Both these factors, population viscosity and the timing of transmission, may also change within-host parasite dynamics. Indeed, in viscous populations and in populations where individuals disperse at the end of infection only, individuals are expected to remain on their patch for longer periods, which may lead to density dependence. Density-dependent parasite growth has been shown in several systems (de Roode et al., 2007; Ebert et al., 2000; Keymer, 1972; Pollitt et al., 2013), and it may affect the relationship between virulence and transmission (Bonneaud et al., 2020), a possibility that remains unexplored. Both population viscosity and the timing of transmission are expected to critically hinge upon host availability during the infection period, as parasites will only effectively disperse when other hosts are attainable. Therefore, host availability during infection is expected to affect virulence, as well as its correlation with transmission.

Here we tease apart the impact of genetic and environmental factors on the relationship between virulence and transmission, using inbred lines of the spider mite *Tetranychus urticae*, a plant macroparasite (Godinho et al., 2020). Spider mites spend their entire life-cycle on their host plants (Helle & Sabelis, 1985), with high intrinsic growth rates, their dispersal (i.e., transmission between hosts), and the damage they inflict resulting in major economic losses in agriculture worldwide (Helle & Sabelis, 1985). This damage, presumably negatively correlated with plant fitness (Fineblum & Rausher, 1995) is visible as chlorotic lesions on the leaf surface. Thus, it can be easily quantified (Mira et al., 2022; Supplementary Figure S1), being a reliable measure of virulence (Noël et al., 2022). Spider mites can transmit to nearby hosts from early on in an infection, or overexploit the host plant before transmission occurs (Bitume et al., 2013; Smitley & Kennedy, 1985). Such transmission depends on many factors, like within-host parasite density, relatedness, and/or the availability of suitable hosts (Bitume et al., 2013; De Roissart et al., 2015). Because spider mites are macroparasites, as a proxy for transmission we used the number of transmitting stages (i.e., adult females (Anderson et al., 1986)), a measure we validate here. We manipulated the opportunity for transmission and the strength of competition of inbred lines of the spider mite *T. urticae* and quantified how this affected the production of transmitting stages and virulence. We assessed whether a relationship between virulence and transmission was determined by genetic differences among lines, and/or by the build-up of density dependence within the host. Additionally, we evaluated whether this relationship was affected by opportunities for transmission during the infection period.

## Materials and methods

### Spider mite inbred lines

*Tetranychus urticae* was collected on different host plants, in Portugal in 2013 (Zélé et al., 2018a), and has since been reared on bean plants (*Phaseolus vulgaris*, variety Prelude), at the University of Lisbon. In October 2015, 50 individuals from six different field populations (total: 300) were collected and mixed to form an outbred population maintained at high densities (>1,000 mites). In October 2016, inbred lines were created from this population by sib mating. This procedure was repeated for 14 generations, ensuring an inbreeding coefficient above 94% (Godinho et al., 2020). Inbred lines allow simultaneous measurement of many individuals of the same (nearly) homozygous genotype, increasing the accuracy of genetic estimates (Godinho et al., 2020). Additionally, given that the original population was outbred, genetic variation across lines is expected to be high and because they were derived from the same population, all lines share the same evolutionary and environmental history (Godinho et al., 2020).

Lines were maintained separately on bean leaf patches in Petri dishes. A subset of 15 inbred lines was transferred to the University of Montpellier in January 2018 and maintained on bean leaves (variety Pongo) in small plastic boxes (255 mm length × 183 mm width × 77 mm height) at optimal conditions (25˚C with a 16:8 L: D cycle, at 60% relative humidity). Under these conditions, egg to adult development takes ca. 11–13 days. These same conditions were kept throughout all experiments.

Prior to the experiments, cohorts of spider mites from each inbred line were created by isolating 40–50 mated females of each line during 48 hr. After 14 days, the resulting mated females (daughters) were used in the experiments. In this species, mated females are the main dispersers (i.e., transmitting stages) and, additionally, the damage inflicted by males is negligible, thus, does not significantly contribute to virulence (Helle & Sabelis, 1985). Not all inbred lines are represented in each experiment due to too few individuals available at the start of the experiment (12–14 lines analyzed).

### Continuous transmission during the infection period

Adult females were randomly assigned to two density treatments (10 or 20 females, respectively), on a 4 cm^2^ bean leaf patch, where they fed and laid eggs for 4 days. Then, adult females were removed, and a photograph of each patch was taken using a Canon EOS 70D camera. The amount of damage inflicted by spider mites was measured using ImageJ and Ilastik 1.3, using a method developed specifically for spider mites ((Mira et al., 2022); Supplementary Figure S1). Host patches unexposed to spider mites were used as a control. The number of eggs laid was registered, as a measure of parasite replication. On day 4, a second leaf patch, uninfected by spider mites, was placed beside the first and connected to it by a 3 × 1 cm Parafilm bridge, thus allowing the dispersal of the emerging adult female offspring (Supplementary Figure S2). The number of adult daughters on the new host patches was checked on days 11, 12, and 13 in blocks 2–4 and on days 12 and 13 in block 1. When there were more than 15 offspring on the new patch, the latter was replaced by a new one, such that uninfected patches were always available. On day 14, we counted the number of adult daughters on the original host patch and on each of the new patches, i.e., the number of transmitting stages produced (potential transmission). The effective transmission was inferred by the cumulative number of females that infected a new host patch. This setup mimics the life cycle of a parasite with continuous transmission during the infection period. There were 5–16 replicates for each inbred line per density treatment, distributed across four blocks.

### Transmission at the end of the infection period

Females of each inbred line were randomly assigned to three density treatments, (5, 10, or 20 founding females), on a 4 cm^2^ bean leaf patch, where they laid eggs for 4 days. They were then removed, a photograph was taken of each leaf to measure the damage inflicted and the number of eggs laid was counted. Fourteen days later, the adult daughters on each patch were counted, i.e., the number of transmitting stages produced (potential transmission). In this setup, transmission would only be possible after this measurement, i.e., at the end of the infection period (Supplementary Figure S2). There were 3–13 replicates for each inbred line per density, distributed across three blocks.

### Statistical analysis

We present correlations between different traits (Table 1): virulence (measured as the damage inflicted) and parasite replication (the number of eggs laid), virulence, and potential transmission (the number of adult daughters, i.e., transmitting stages). Additionally, to test whether density dependence rises from the negative effects of intraspecific competition on the survival of juvenile stages, we check the correlation between virulence and juvenile mortality (i.e., the proportion of eggs that did not develop into adults). We also validate our measure of transmission by assessing the correlation between the number of transmitting stages (potential transmission) and the number of females colonizing novel host patches (effective transmission). To test genetic correlations, we consider total values per host patch, as theoretical predictions are tailored to such population values (Anderson & May, 1982; Anderson et al., 1986) and most experimental studies on this topic have used them (de Roode et al., 2008; Doumayrou et al., 2013; Mackinnon & Read, 1999). Note however that using per capita data would not modify conclusions on correlations, as density would be present in both numerator and denominator.

**Table 1.** Genetic and environmental correlations between traits measured.

Section	Traits	Experiment	DIC		Across densities		Density 5		Density 10		Density 20	
			No density	Density	Genetic correlation	Environmental correlation	Genetic correlation	Environmental correlation	Genetic correlation	Environmental correlation	Genetic correlation	Environmental correlation
a	Transmitting stages vs. transmission	Continuous	3,632*	3,632	HPDI: 0.94, 0.99; rg = 0.99	HPDI: 0.99, 0.99; re = 0.99	na	na	HPDI: 0.48, 0.99; rg = 0.99	HPDI: 0.99, 0.99; re = 0.99	HPDI: 0.81, 0.99; rg = 0.99	HPDI: 0.79, 0.99; re = 0.99
b	Virulence vs. parasite replication	Continuous	4,956*	5,007	HPDI 0.91,0.94; rg = 0.93	HPDI: 0.49, 0.65; re = 0.57	na	na	HPDI: −0.99, 0.80; rg = 0.04	HPDI: 0.30, 0.54; re = 0.40	HPDI: 0.96, 0.99; rg = 0.97	HPDI: 0.24, 0.58; re = 0.42
		End	6,109*	6,182	HPDI: 0.79, 0.99; rg = 0.99	HPDI: 0.51, 0.65; re = 0.59	HPDI: −0.96, 0.99; rg = 0.09	HPDI: 0.07, 0.40; re = 0.25	HPDI: −0.97, 0.99; rg = 0.09	HPDI: 0.28, 0.57; re = 0.43	HPDI: 0.92, 0.99; rg = 0.99	HPDI: 0.30, 0.61; re = 0.51
c	Virulence vs transmitting stages	Continuous	4,244	4,240*	HPDI: 0.83, 0.99; rg = 0.99	HPDI: −0.24, 0.03; re = −0.09	na	na	HPDI: 0.51, 0.99; rg = 0.99	HPDI: 0.23, 0.64; re = 0.39	HPDI: 0.44, 0.99; rg = 0.99	HPDI: 0.15, 0.24; re = 0.16
		End	5,564*	5,585	HPDI: -0.84, 0.99; rg = 0.09	HPDI: −0.29, −0.08; re = -0.15	HPDI: −0.99, 0.99; rg = 0.09	HPDI: 0.13, 0.45; re = 0.28	HPDI: −0.99, 0.97; rg = −0.01	HPDI: −0.10, 0.25; re = 0.02	HPDI: −0.99, −0.13; rg = −0.99	HPDI: −0.45, −0.06; re = −0.27
d	Virulence vs. juvenile mortality	Continuous	1,882	1,869*	HPDI: -0.99, 0.05; rg = -0.81	HPDI: 0.03, 0.29; re = 0.17	na	na	HPDI: −0.97, 0.28; rg = −0.72	HPDI: −0.07, 0.35; re = 0.22	HPDI: −0.94, 0.58; rg = −0.60	HPDI: −0.06, 0.31; re = 0.14
		End	2698	2649*	HPDI: -0.43, 0.99; rg = 0.09	HPDI: 0.25, 0.47; re = 0.38	HPDI: −0.97, 0.99; rg = 0.08	HPDI: 0.01, 0.34; re = 0.2	HPDI: −0.85. 0.98; rg = 0.08	HPDI: 0.15, 0.47; re = 0.28	HPDI: 0.71, 0.99; rg = 0.98	HPDI: 0.27, 0.60; re = 0.44

*Note*. Genetic (rg) and environmental (re) correlations—extracted from the genetic and residual error structure of the models, respectively, —were measured between (a) the production of transmitting stages (adult daughters) and transmission, and between virulence and (b) parasite replication, (c) the production of transmitting stages (adult daughters), and (d) juvenile mortality (i.e., the proportion of eggs that did not develop into adults). All traits were measured per host (as the sum of the contribution of all females initially placed on the leaf). The deviance information criterion (DIC) for models with and without density included is shown. Models with the lowest DIC or the simplest model (when there was no difference in DIC values), are identified using an *. Highest posterior density intervals (HPDIs) and correlation coefficients (rg and re) are shown for the models including all data, and separately for each of the different densities. Intervals that do not include zero are shown in bold. Experiment: (1) continuous: continuous transmission; (2) end: transmission at the end of the infection period.

Genetic and environmental correlations were measured using a multiresponse generalized linear mixed model fitted with an MCMCglmm (package MCMCglmm; (Hadfield, 2010)). Genetic correlations were determined by including the identity of the line as a random factor in each model and assessing the highest posterior density interval (HPDI) of the genetic (G) structure of the model, which represents the (co)variances between the two traits evaluated across inbred lines (Hadfield, 2010). Environmental correlations were obtained by assessing the HPDI of the residual (R) structure in the same model (Hadfield, 2010). Effects were considered significant when the HPDI did not include zero (Hadfield, 2010). The effect of initial density on correlations was assessed by comparing the deviance information criterion (DIC) of a model not including density with that of a model including density as a covariate (i.e., making it a function-value trait; (Gomulkiewicz et al., 2018)). Models were considered different when the difference in DIC was higher than 2. Additionally, we report the genetic and environmental correlations for each density level separately (Table 1). We also tested if the relationship between virulence and transmission might be quadratic or saturating, as predicted by theoretical models (Alizon et al., 2009; Anderson & May, 1982), by comparing the DIC of models with the number of transmitting stages produced as the response variable with virulence included as a linear term, with models that also included the quadratic and/or square-rooted terms for virulence, respectively.

To measure genetic variance for traits (variance among inbred lines) and the effect of density on this variance we used per capita values, by dividing the value for each host patch by the initial density of adult females (Supplementary Figure S3, Supplementary Table S1). Note that here we did not use population values because heritability measures concern individuals, not population traits. We then applied generalized linear mixed models fitted with a Markov Monte Carlo Chain approach (Hadfield, 2010) with per capita trait values as the response variable and density included in the model as a fixed factor and inbred line as a random factor. Broad-sense heritability, $\;\;\;H^{2}=\frac{Var\;\left((G)\right)}{Var\;\left(G\right)+Var\;\left((E)\right)}$(Falconer & Mackay, 1996) and the corresponding confidence intervals were extracted from the abovementioned models for each trait. Because heritability is a proportion between 0 and 1, and thus the HPDI is bounded to fluctuate above 0, instead, we determined if there was significant genetic variation by (a) assessing the 95% HPDI of the genetic component of each model (i.e., variance among inbred lines) and (b) comparing, for each trait, the DIC of a model including inbred line as a random factor with that of a model without inbred line as a random factor (Henriques et al., 2021).

All models initially included 300,000 iterations, with a burn-in of 10,000 iterations, thinning of 100 and a flat prior: for generalized linear mixed models (GLMMs) (to assess genetic variance), *V* = 1 and nu = 0.002, assigning a weak informative variance (*V*) and a low degree of belief (nu) (Gelman, 2006); for multiresponse GLMMs (to assess trait correlations), *V* = matrix(c(1,0,0,1), ncol = 2, nrow = 2) and nu = 0.002, meaning no covariance between traits, in accordance with the null hypothesis of no correlation. Additionally, we ran the models using an uninformative prior based on an inverse-gamma distribution (*V* = diag(2)*(0.002/1.002), nu = 1.002), which gave similar results (not shown) (Gelman, 2006). These priors were used to allow the hyperparameter values to reflect a reasonable range of values for the traits in question, without any previous information about them or their covariance. All models were checked for convergence with a stationary test using the heidel.diag function and for autocorrelations in the Markov chain within fixed and random terms using the autocorr.diag function (package coda (Plummer et al., 2006)). When models failed one of these tests, the number of iterations was increased to 500,000 or 700,000 and the burn-in to 20,000 or 50,000. All figures were produced with the ggplot2 package in R and the regressions fitted with the geom_smooth function (Wickham & Winston, 2016).

## Results

### Validation of our measure of transmission

**Figure 1.**
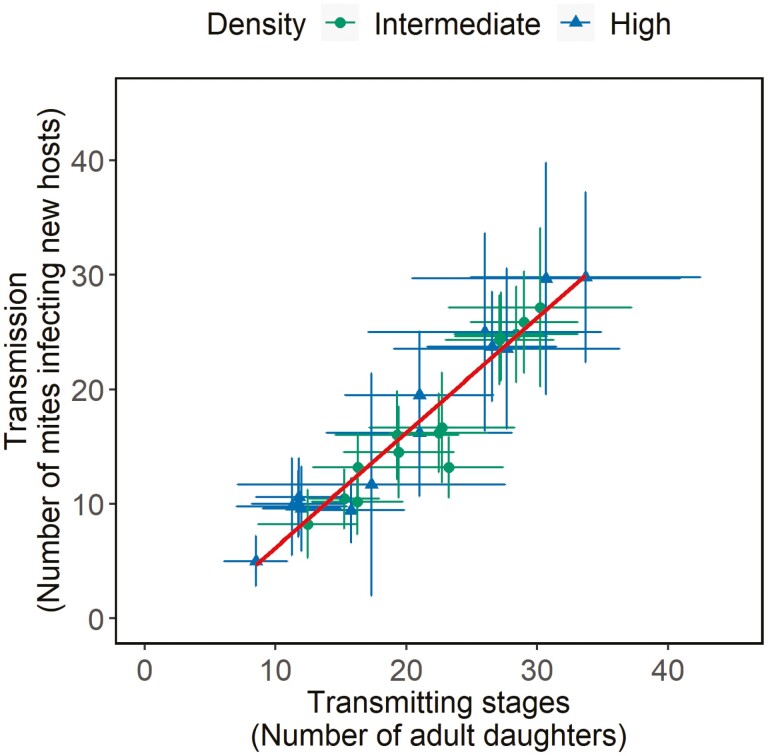
Correlation between transmitting stages and transmission. The number of daughters produced (transmitting stages) and transmission (the number of mites infecting new hosts) for inbred lines of *T. urticae* infesting hosts at different starting densities (10 females = green circles; 20 females = blue triangles) with the possibility of continuous transmission; dots are the mean for each inbred line ± standard error; the regression (fitted with geom_smooth function) is represented in red.

We found a positive correlation, both environmental (i.e., among residuals of the two variables, *r*_*e*_ = 0.99) and genetic (i.e., among the factor “inbred line” for the two variables, *r*_*g*_ = 0.99), between the number of transmitting stages produced (i.e., adult daughters) and transmission to uninfected hosts (Table 1a; Figure 1), indicating that the former is a good proxy for the latter.

### Genetic variation for replication rate, virulence, and transmission

To observe genetic correlations among parasite traits, genetic variance for these traits must be present in the population. Thus, we quantified genetic variance for virulence (% of the damage inflicted), parasite replication (number of eggs), and transmission (the number of transmitting stages) among the *T. urticae* inbred lines (Supplementary Figure S3), using per capita measurements, which allows the determination of broad sense heritability, *H*^2^ (Supplementary Table S1, Supplementary Figures S4 and S5). We found significant genetic variation for all traits, as the 95% HPDI of the genetic component in the models did not include 0 (Supplementary Table S1) and the DIC of models including the inbred line as a random factor were lower than those excluding it (Supplementary Table S2). Additionally, all traits were affected by the initial density of founding females on the host patch (Supplementary Table S3), but not in a similar way. Indeed, the transmission of all lines decreased with density, whereas for parasite replication and virulence the pattern was line-specific (Supplementary Figure S3).

### Correlation between virulence and parasite replication

**Figure 2.**
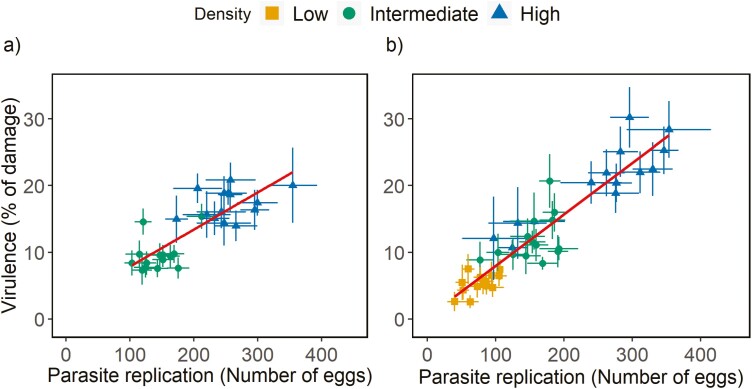
Correlation between parasite replication and virulence in inbred lines of *T. urticae* infecting a host patch. The total damage inflicted on the host patch (i.e., virulence) and the total number of eggs laid (parasite replication) were measured in a set-up with (A) continuous transmission during the infection period and (B) transmission at the end of the infection period. Colors represent different densities (5 females = yellow squares; 10 females = green circles; and 20 females = blue triangles); dots are the mean for each inbred line ± standard error; because there is no effect of density on these correlations, regressions (in red) are represented across densities (fitted with the geom_smooth function).

We found positive genetic and environmental correlations between virulence (% of the damage inflicted) and parasite replication (number of eggs laid) both when transmission occurred continuously during the infection period and when transmission was restricted to the end of the infection period (Table 1b, Figure 2). These relationships were not affected by the initial parasite density (Table 1b).

### Correlation between virulence and transmission

**Figure 3.**
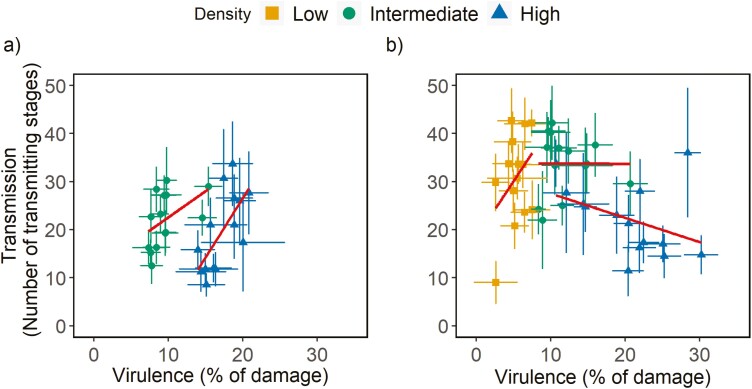
Correlation between virulence and transmission in inbred lines of *T. urticae* infecting a host patch. The total damage inflicted on the host patch (i.e., virulence) and the total number of transmitting stages (transmission) were measured in a set-up with (A) continuous transmission during the infection period and (B) transmission at the end of the infection period. Colors represent different densities (5 females = yellow squares; 10 females = green circles; and 20 females = blue triangles); dots are the mean for each inbred line ± standard error; because there are differences among densities, regressions (in red) are represented for each density (fitted with the geom_smooth function).

**Figure 4.**
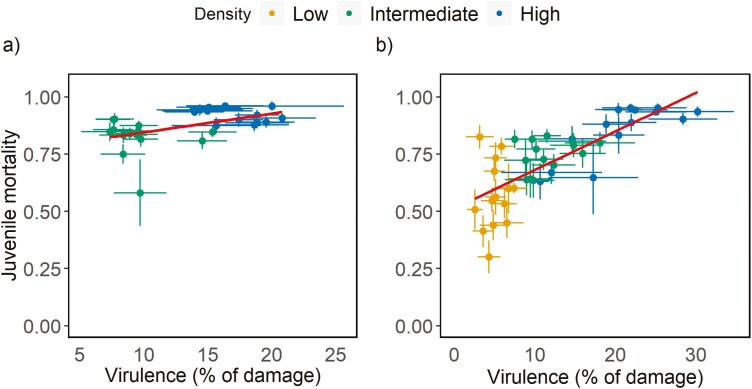
Correlation between virulence and juvenile mortality in inbred lines of *T. urticae* infecting a host patch. The damage inflicted on the host patch (i.e., virulence) and juvenile mortality (i.e., the proportion of eggs that did not develop into adults) were measured in a set-up with (A) continuous transmission during the infection period and (B) transmission at the end of the infection period. Densities are coded in yellow squares (5 females), green circles (10 females), and blue triangles (20 females); dots are the mean for each inbred line ± standard error; regressions (fitted with geom_smooth function) are shown in red.

When transmission was continuous, there was no overall environmental or genetic correlation between virulence and transmission across densities. However, at each density (intermediate and high), we found positive environmental and genetic correlations between virulence and transmission (Table 1c; Figure 3A). In this scenario, selection on virulence will directly affect transmission, therefore parasite fitness is expected to increase with virulence. Accordingly, we did not find any evidence for a quadratic or saturating relationship between virulence and transmission (DIC 2,026.7 and 2,026.6, respectively, as compared with the linear model 2,026.4). In contrast, when transmission was restricted to the end of the infection, we found a negative environmental correlation between virulence and transmission, but no genetic correlation between these traits (Table 1c, Figure 3B). Furthermore, although there was no quadratic nor saturating relationship between these traits (DIC 2,691.1 and 2,691.1, respectively, as compared with the linear model 2,690.9), the direction of the correlation between virulence and transmission changed from positive at low density, to absent at intermediate density and negative at high density (Table 1c). This density dependence pertains from the negative effects of intraspecific competition at high densities, which is confirmed by a positive environmental correlation between virulence and juvenile mortality at all densities, which was not the case in the scenario with continuous transmission (Table 1d, Figure 4). In this scenario, with transmission at the end of the infection period, virulence is not genetically correlated with transmission, but high virulence leads to strong density dependence within hosts, independently of the genotype considered.

## Discussion

In this study, we show that opportunities for transmission shape the relationship between virulence and transmission, a key component of parasite fitness. We found a positive genetic correlation between virulence and transmission when the latter was continuous during infection, independently of the initial parasite density. In contrast, when transmission was restricted to the end of the infection period, the relationship between these traits no longer had a genetic basis, but instead, was modulated by density dependence: beyond a certain level of virulence, at high initial densities of mites, despite an increase in replication (i.e., number of eggs laid), within-host competition prevents most offspring from becoming adult, such that highly virulent genotypes produce fewer adult daughters. Evidence for density dependence has been found in this (De Roissart et al., 2015; Rotem & Agrawal, 2003), and other host-parasite systems, including microparasites (de Roode et al., 2007; Ebert et al., 2000; Pollitt et al., 2013).

The positive genetic correlation between virulence and transmission, found when parasites could transmit during the infection period, suggests that there will be selection for high virulence. If this is the case, why then do we still find genetic variance for this trait? We propose three nonmutually exclusive hypotheses. First, *T. urticae* is a generalist parasite, hence it may frequently shift among hosts, and optimal virulence may vary with the host species (Gandon, 2004; Magalhães et al., 2014; Zélé et al., 2018b). Furthermore, different host plant species, as well as different individuals of the same host plant, may harbor different heterospecific parasites (Dinnage et al., 2012; Wimp et al., 2004). Coinfections with other parasites are expected to select for different levels of virulence depending on the direction and mechanism of the interaction (Alizon et al., 2013; Choisy & de Roode, 2010; Clay & Rudolf, 2019). Finally, transmission within the infection period relies on the occurrence of hosts to which parasites can transmit. This may not always be possible, as uninfected hosts may be locally absent or become rapidly infected (Crossan et al., 2007; Hochberg, 1991). Mites are known to base their decision to leave a host on the perception of cues (e.g., volatiles) from hosts in the environment, including their infection status (Kiedrowicz et al., 2017; Pallini et al., 1997). Therefore, in the absence of uninfected hosts, mites may remain on their host until they overexploit it, which will eventually result in them switching to a parasite life cycle with transmission occurring only at the end of the infection period.

In the latter scenario, when infection was restricted to the end of the infection, we found no genetic correlation between virulence and transmission. Instead, we found a positive environmental correlation at low density, which became negative at high density, leading to a peak of transmission at intermediate levels of virulence, as predicted by theory (Anderson & May, 1982). However, this relationship between virulence and transmission was not determined by differences among inbred lines, hence it was purely environmental. The fact that the negative correlation between virulence and transmission disappears when transmission occurs continuously during the infection further supports the hypothesis that density dependence modulates the peak of transmission at intermediate levels of virulence only when transmission occurs at the end of the infection period. This effect of the timing of transmission may also explain the mixed empirical evidence for the occurrence of the virulence transmission trade-off, as continuous transmission will only be relevant for some parasite life-cycles and/or experimental designs (Acevedo et al., 2019).

Such modulation of epidemiological trade-offs by the environment has been found in host traits in another system (Hall et al., 2012). Also, previous work has shown that limited opportunities for transmission can select for lower virulence, ensuring a reduction in kin competition and/or host depletion (Lion & Boots, 2010; Wild et al., 2009). In line with this, parasite transmission in more structured populations has been shown to select for lower virulence (Berngruber et al., 2013; Boots & Mealor, 2007; Kerr et al., 2006). We do not find this in our experiment. In fact, at all densities, virulence and parasite replication (i.e., the number of eggs) were positively correlated. However, at high densities, we observed high juvenile mortality (Figure 4). Therefore, genotypes that induce more harm to their host will not necessarily produce more transmitting stages, which explains the absence of a positive genetic correlation between virulence and transmission. The negative environmental correlation found between these traits at high densities may simply be a by-product of density dependence: fewer transmitting stages are produced in plant patches that are more damaged by parasites. Possibly, spider mites are not selected to thrive in highly competitive environments, hence they still produce as many eggs as possible (a trait selected in low-competition environments) but then these individuals die due to the absence of resources. Alternatively, spider mites may have been selected in saturated environments, but in this case, we would expect them to maximize their competitive ability, rather than resource consumption, which would also not lead to more virulent genotypes producing more transmitting stages. In any case, under this scenario, given the breakdown of the genetic correlation between virulence and transmission, selection on virulence may not affect transmission. In other words, the evolution of virulence may not affect parasite fitness, given that parasite fitness includes both within-host (parasite replication) and between-host (transmission, here the number of transmitting stages) traits (Alizon & Michalakis, 2015; Anderson & May, 1982). Thus, variance for virulence in parasite populations, as observed in this (Supplementary Figure S3) and other systems (Dutta et al., 2021; Little et al., 2008; Mackinnon & Read, 1999a) may be maintained, instead, by the heterogeneity pertaining to host availability, variability, and connectivity (Boots & Mealor, 2007; Lion & Boots, 2010; Thrall & Burdon, 2003). Moreover, the (co)evolution of host traits, such as resistance, may create temporal heterogeneity for parasites, contributing to the maintenance of variance for virulence in parasite populations (Lambrechts et al., 2006).

Our results support the prediction that high virulence is advantageous in the beginning of the infection period, when many uninfected hosts are available, but this advantage may be lost later on when fewer hosts are available (Day, 2003). Indeed, in our study, we find a positive correlation between virulence and transmission, both under continuous transmission and transmission at the end of the infection period, but in the latter case only when parasite density is low, which should typically occur at the beginning of epidemics. At high density, though, this correlation is negative, which suggests that reduced transmission opportunities could result in lower parasite virulence, if parasites respond plastically to the current environment, as has been shown in viruses and *Plasmodium* (Leggett et al., 2013; Reece et al., 2008). Besides affecting the relationship between virulence and transmission, host availability may also affect the length of infection, the other key component of parasite fitness (Anderson & May, 1982), since early transmission may slow host exploitation. However, our design did not allow testing this hypothesis.

In sum, we show that virulence is genetically correlated with the transmission. However, this key assumption of most theoretical models of the evolution of virulence only holds under continuous transmission. Instead, selection for higher virulence in this system is expected to disappear under limited parasite transmission due to within-host density dependence. This suggests that this fundamental difference in the parasite life cycle, i.e., continuous transmission vs. transmission at the end of the infection period, has key implications for virulence evolution. Additionally, it highlights that the effect of within-host density dependence may hinge upon the parasite life cycle. Indeed, whereas for parasites that transmit at the end of the infection period (e.g., obligate killers), within-host density dependence may underlie the predicted optimal transmission at intermediate virulence levels, for parasites that transmit continuously, it may only come into play later in the epidemic, when uninfected hosts become limited. In any case, our results demonstrate that only by including the whole parasite life-cycle in experimental set-ups is it fully possible to understand the ecology and evolution of virulence (Alizon & Michalakis, 2015).

## Supplementary Material

qrac008_suppl_Supplementary_Material

## Data Availability

Data are deposited in Dryad: https://doi.org/10.5061/dryad.4xgxd25dv.
