# Supplementary material for: Limited host availability disrupts the genetic correlation between virulence and transmission
Source: Evol Lett. 2023 Jan 31;7(1):58–66. doi: 10.1093/evlett/qrac008 (PMC10091498; doi:10.1093/evlett/qrac008)
Supplement: qrac008_suppl_Supplementary_Material [file qrac008_suppl_supplementary_material.pdf]

Supplementary Information for

“Limited host availability disrupts the genetic correlation between virulence and transmission”

Authors

Diogo P. Godinho*[1], Leonor R. Rodrigues[1], Sophie Lefevre[2], Laurane Delteil[2], André F. Mira[1], Inês R. Fragata[1], Sara Magalhães[1,3]† and Alison B. Duncan[2]†

Affiliations

1. cE3c: Centre for Ecology, Evolution, and Environmental Changes, Faculty of Sciences, University of Lisbon, Edifício C2, 3º piso, 1749-016 Lisboa, Portugal

2. Institut des Sciences de l’Évolution, Université de Montpellier, CNRS, IRD, EPHE, CC065, Place Eugène Bataillon, 34095 Montpellier Cedex 05, France

3. Departamento de Biologia Animal, Faculdade de Ciências da Universidade de Lisboa

*Correspondence to: diogoprinogodinho@gmail.com

†(equal last author)

This file includes:

Figure S1. Example of a host patch cut from a bean plant (*Phaseolus vulgaris*) upon image acquisition and after software output.

Figure S2. Experimental set-ups used.

Figure S3. Genetic variance for traits measured.

Figure S4. Density of the distribution of the estimated values of heritability for the traits measured with continuous transmission.

Figure S5. Density of the distribution of the estimated values of heritability for the traits measured with transmission at the end of the infection.

Table S1. Broad-sense heritability and genetic variance of the measured traits.

Table S2. The effect of genetic variation on the measured traits.

Table S3. The effect of the initial density of female spider mites on traits measured.

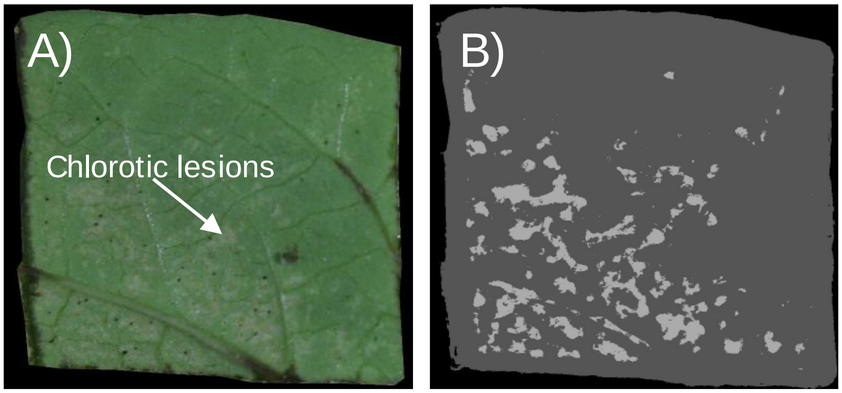

Figure S1. Example of a host patch cut from a bean leaf (*Phaseolus vulgaris*) upon image acquisition and after software output. A) Leaf damage (chlorotic lesions) caused by *T. urticae* feeding. B) The photograph is transformed into a simple segmentation image using Ilastik 1.3. Areas of leaf that are damaged are shown in light grey and correspond to our measure of virulence.

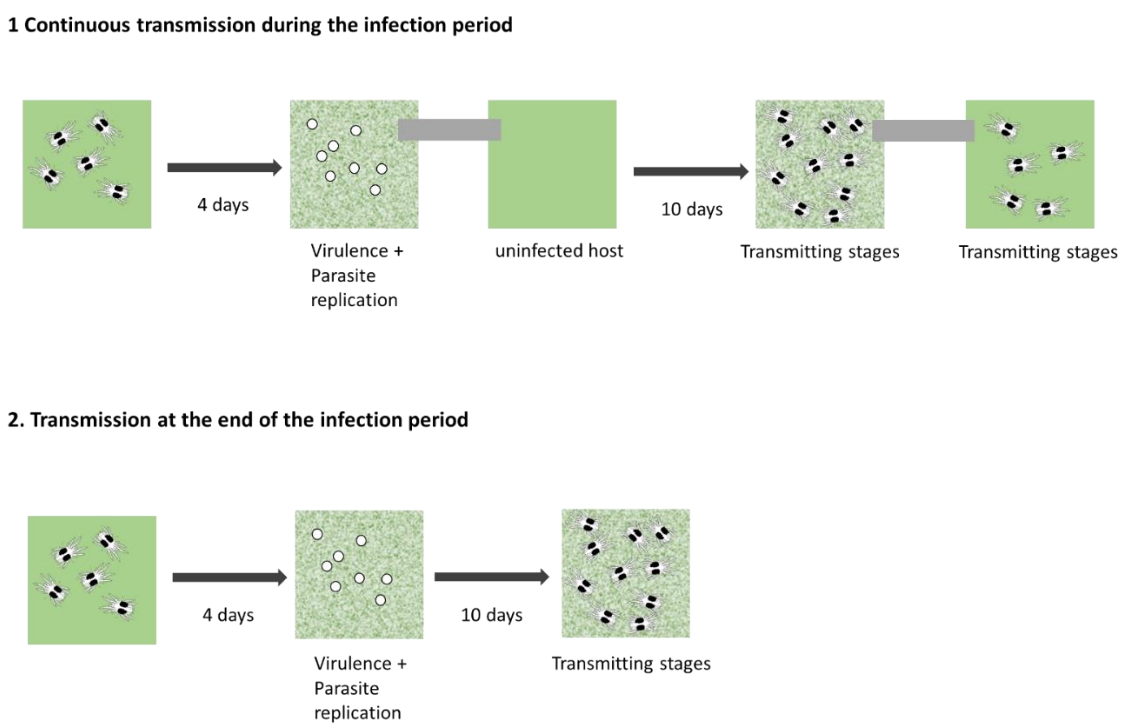

Figure S2. Experimental set-ups employed. Schematic representation of the experimental set-ups and the traits that were measured in each experiment. Day zero shows five spider mites on a healthy four cm^2 leaf patch (treatments with starting densities of 10 and 20 are not shown). On day four, virulence (mottled white areas on leaf patches) and parasite replication (i.e. number of eggs) were measured in experiments one and two. The number of transmitting stages (i.e adult daughters) was measured 14 days after mite installation. In experiment one, transmission to an uninfected host patch was possible from day four to day 14 across a Parafilm bridge (grey rectangle linking leaf patches).

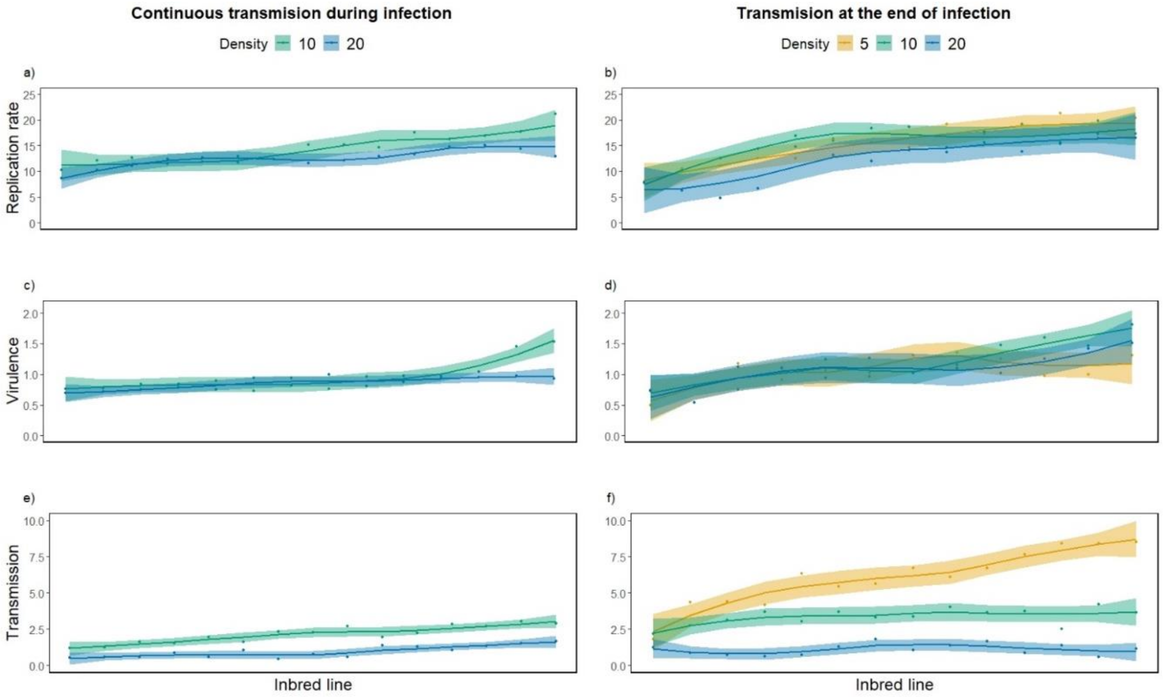

Figure S3. Variation in the measured traits among inbred lines. Variation for replication rate (number of eggs laid; a and b), virulence (% of damage inflicted; c and d), and transmission (the number of transmitting stages; e and f), measured per capita in the different experiments (‘Continuous transmission’ experiment – a, c, and e; ‘Transmission at the end of infection’ experiment – b, d, and f). Dots represent the mean values per line, at each starting density (yellow = 5 females; green = 10 females; blue = 20 females). The inbred lines are shown on the figures in rank order (according to mean values across densities), and this may change between panels. The shape of the curve for each density was calculated using a polynomial regression fitted with the geom_smooth function.

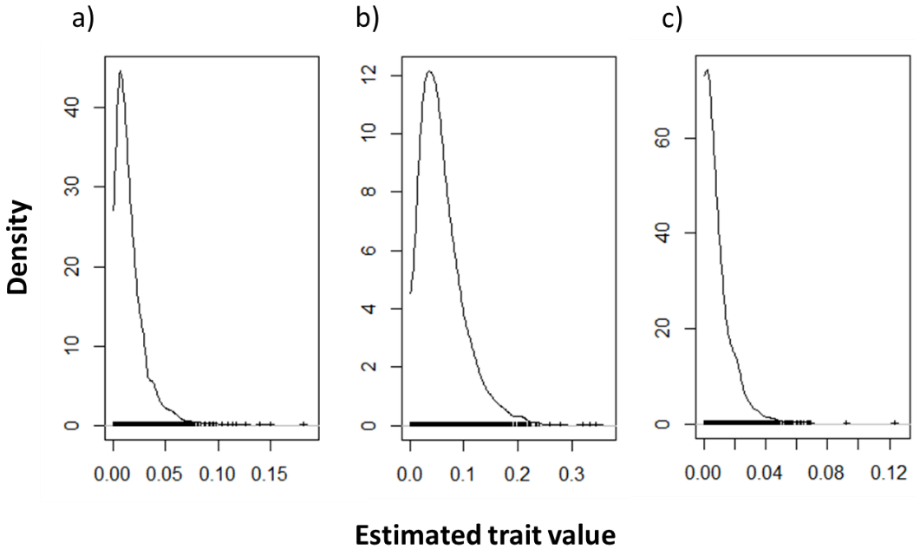

Figure S4. Density of the distribution of the estimated values of heritability for the traits measured with continuous transmission. Distribution of the estimates for a) replication rate (number of eggs laid), b) virulence (% of damage inflicted) and c) transmission (the number of transmitting stages), measured per capita in experiment 1 - 'Continuous transmission'.

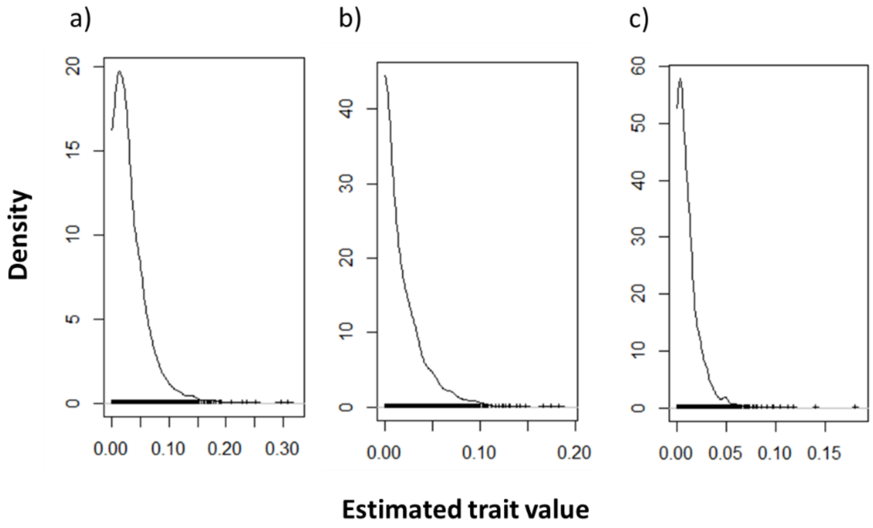

Figure S5. Density of the distribution of the estimated values of heritability for the traits measured with transmission at the end of the infection. Distribution of the estimates for a) replication rate (number of eggs laid), b) virulence (% of damage inflicted) and c) transmission (the number of transmitting stages), measured per capita in experiment 2 - 'Transmission at the end of infection'.

Table S1. Broad-sense heritability and genetic variance of the measured traits. Broad-sense heritability and genetic variance of per capita parasite replication, virulence and transmission (transmitting stages) measured in the different experiments. 95% highest posterior density intervals (HPDI) intervals are shown for the heritability and the genetic component of the variance (i.e. among line variation) of each trait. Experiment: 1) continuous: continuous transmission; 2) end: transmission at the end of the infection period.

**Trait**	**Experiment**	**Heritability**		**Genetic variance**	
		estimate	95% HPDI	estimate	95% HPDI
**Parasite replication**	continuous	0.02	0.001, 0.04	5.61	1.16, 11.49
	end	0.04	0.001, 0.09	12.20	3.03, 24.62
**Virulence**	continuous	0.06	0.001, 0.14	0.03	0.001, 0.06
	end	0.03	0.001, 0.07	0.02	0.001, 0.07
**Transmission**	continuous	0.07	0.001, 0.03	0.15	0.001, 0.36
	end	0.01	0.001, 0.04	0.81	0.12, 1.82

Table S2. The effect of genetic variation on the measured traits. Per capita parasite replication (number of eggs laid), virulence and transmission (transmitting stages), were modelled with and without inbred line as a random factor. Deviation information criterion (DIC) of the models (MCMCglmm package) are shown, with the best fit models represented in bold. Experiment: 1) continuous: continuous transmission; 2) end: transmission at the end of the infection period.

**Trait**	**Experiment**	**Model**	**DIC**
**Parasite replication**	continuous	trait ~ -1 + Density + random= Block	2039
		**trait ~ -1 + Density + random= Line + Block**	**1999**
	end	trait ~ -1 + Density + random= Block	2713
		**trait ~ -1 + Density + random= Line + Block**	**2651**
**Virulence**	continuous	trait ~ -1 + Density + random= Block	473
		**trait ~ -1 + Density + random= Line + Block**	**453**
	end	trait ~ -1 + Density + random= Block	993
		**trait ~ -1 + Density + random= Line + Block**	**989**
**Transmission**	continuous	trait ~ -1 + Density + random= Block	1131
		**trait ~ -1 + Density + random= Line + Block**	**1119**
	end	trait ~ -1 + Density + random= Block	1883
		**trait ~ -1 + Density + random= Line + Block**	**1857**

Table S3. The effect of the initial density of female spider mites on traits measured. Per capita parasite replication (number of eggs laid), virulence and transmission (transmitting stages), were modelled with and without initial parasite density as a covariate. Deviation information criterion (DIC) of the models (MCMCglmm package) are shown, with the best fit models represented in bold. Experiment: 1) continuous: continuous transmission; 2) end: transmission at the end of the infection period.

Trait	Experiment	Model	DIC
**Parasite replication**	continuous	trait ~ -1 + random= Line + Block	2013
		**trait ~ -1 + Density + random= Line + Block**	**1999**
	end	trait ~ -1 + random= Line + Block	2665
		**trait ~ -1 + Density + random= Line + Block**	**2651**
**Virulence**	continuous	trait ~ -1 + random= Line + Block	461
		**trait ~ -1 + Density + random= Line + Block**	**453**
	end	trait ~ -1 + random= Line + Block	998
		**trait ~ -1 + Density + random= Line + Block**	**989**
**Transmission**	continuous	trait ~ -1 + random= Line + Block	1189
		**trait ~ -1 + Density + random= Line + Block**	**1119**
	end	trait ~ -1 + random= Line + Block	2076
		**trait ~ -1 + Density + random= Line + Block**	**1857**
